# How do past global experiences of coal phase-out inform China’s domestic approach to a just transition?

**DOI:** 10.1007/s11625-023-01312-5

**Published:** 2023-04-12

**Authors:** Vigya Sharma, Julia Loginova, Ruilian Zhang, Deanna Kemp, Guoqing Shi

**Affiliations:** 1 Centre for Social Responsibility in Mining, Sustainable Minerals Institute, The University of Queensland, Brisbane, Australia; 2 National Research Centre for Resettlement (NRCR) and Asian Research Centre, Hohai University, Nanjing, China

**Keywords:** China, Just transitions, Coal phase-out, Energy transition

## Abstract

China produces nearly half of the world’s coal and more than half of the global coal-fired electricity. Its CO_2_ emissions are higher than the combined volumes of the next three world regions—the US, Europe, and India. China has announced a net-zero commitment by 2060. This timeline creates enormous pressure to maintain energy security while phasing down coal use. Despite the localized nature of China’s coal production with nearly 80% of its thermal coal industry concentrated in four provinces, the dependencies are complex and extensive. Large-scale changes to energy systems will result in a range of social, cultural, and economic disruptions across China’s urban, rural, and remote regions. This paper examines experiences with coal transitions in other jurisdictions and considers implications for China. We examine the drivers, successes, and failures of coal phase-down in Germany, Poland, Australia, the UK, and the US. Despite significant differences in scale and complexity, these experiences offer important insights for China as it works to meet its climate commitments.

## Introduction

The People’s Republic of China (China) is the world’s largest greenhouse gas emitter owing to its high energy demand—a demand that is overwhelmingly met by coal (IEA 2021). The Chinese government has committed to reaching net-zero emissions by 2060 (NCSC 2021). While there has been a rapid increase in China’s hydropower, wind and solar energy capacity in the last three decades, coal phase-down has been slow (Climate Action Tracker 2022). China’s decarbonization challenge is enormous and multi-dimensionally complex (Li 2010; Zhang and Chen 2022) with energy security concerns and supply uncertainties prolonging China’s reliance on coal (Climate Action Tracker 2022). As renewable energy becomes more reliable, affordable, and grid-integrated, China intends to pursue the gradual closure of coal mines and coal-fired power plants and has introduced several institutional incentives to support the energy transition process (Cao et al. 2016; Dong et al. 2022; Zheng et al. 2014). How these incentives affect the coal sector, particularly at the sub-national and local levels, and what kind of social impacts may emerge consequently calls for careful analysis.

This review paper seeks to address the gap in available knowledge about the social impacts that may result from coal phase-down in China. It has two main objectives: first, to learn lessons from elsewhere. China is not the first jurisdiction to pursue a large-scale transition in its energy system. Existing literature offers comprehensive accounts of these transitions in other jurisdictions, a large majority of which are in the Global North (Caldecott et al. 2017; Diluiso et al. 2021; Ohlendorf et al. 2022). Coal transitions in Germany, the United Kingdom (UK), Australia, Poland, and the United States (US), for instance, have been well documented and widely studied (Brauers and Oei 2020; Braunger and Walk 2022; Della Bosca and Gillespie 2018; Oei et al. 2020a, b). These experiences are context-specific to the socio-economic, cultural and institutional conditions in which they occurred, and are in many ways dissimilar to the Chinese context (Huang et al. 2021). Nonetheless, the opportunity exists to examine these experiences—successful and failed—in light of China’s climate commitments.

Second, there is growing attention to the idea of a “just” energy transition. A just transition represents a vision for a participatory, dialog-driven change process that prioritizes social justice and addresses social vulnerabilities induced by large-scale industrial change (Heffron 2021). Coined in the 1970s by the trade union movement in North America, the idea of a just transition has expanded, and while social justice sits at the core, there is no single conception of, or approach to, a just transition (Błachowicz et al. 2021; Sovacool et al. 2022; Snyder 2018). Energy transition policy and its focus on justice vary depending on the geographical, social, economic, cultural, and political context in which they are being applied. There is limited research on how the idea of a just transition might apply in China, given the scale and complexity of its energy transition, and the nature of its political, economic and justice systems. Drawing on current academic framings of just transitions and coal phase-out experiences elsewhere, this paper examines the processes and attributes that may help relate the idea of a just transition to China’s coal phase-down.

The paper proceeds on the premise that there is an opportunity to synthesize diverse experiences from other jurisdictions and offer valuable lessons for China as it transitions away from coal. These lessons provide a useful reminder that coal transitions are messy and deeply complex (Wang and Lo 2022). Drawing together experiences from elsewhere and considering their applicability to China complements calls for transition approaches that are culturally sensitive and consider China’s existing political economy structures (Huang et al. 2021). It also proceeds in recognition of the broader geopolitical context of Russia’s war in Ukraine. The war has driven price volatility and energy insecurity and affordability and has prompted a rapid re-consideration of coal phase-out among energy-insecure countries across the Global North (Halper 2022). China’s energy transition is intertwined with these developments and is likewise vulnerable to uncertainty and instability. The findings from this paper are timely as China has an important leadership role to play in the global energy discourse through its ambitious climate policymaking.

### A brief note on the paper’s methodological approach

**Fig. 1:**
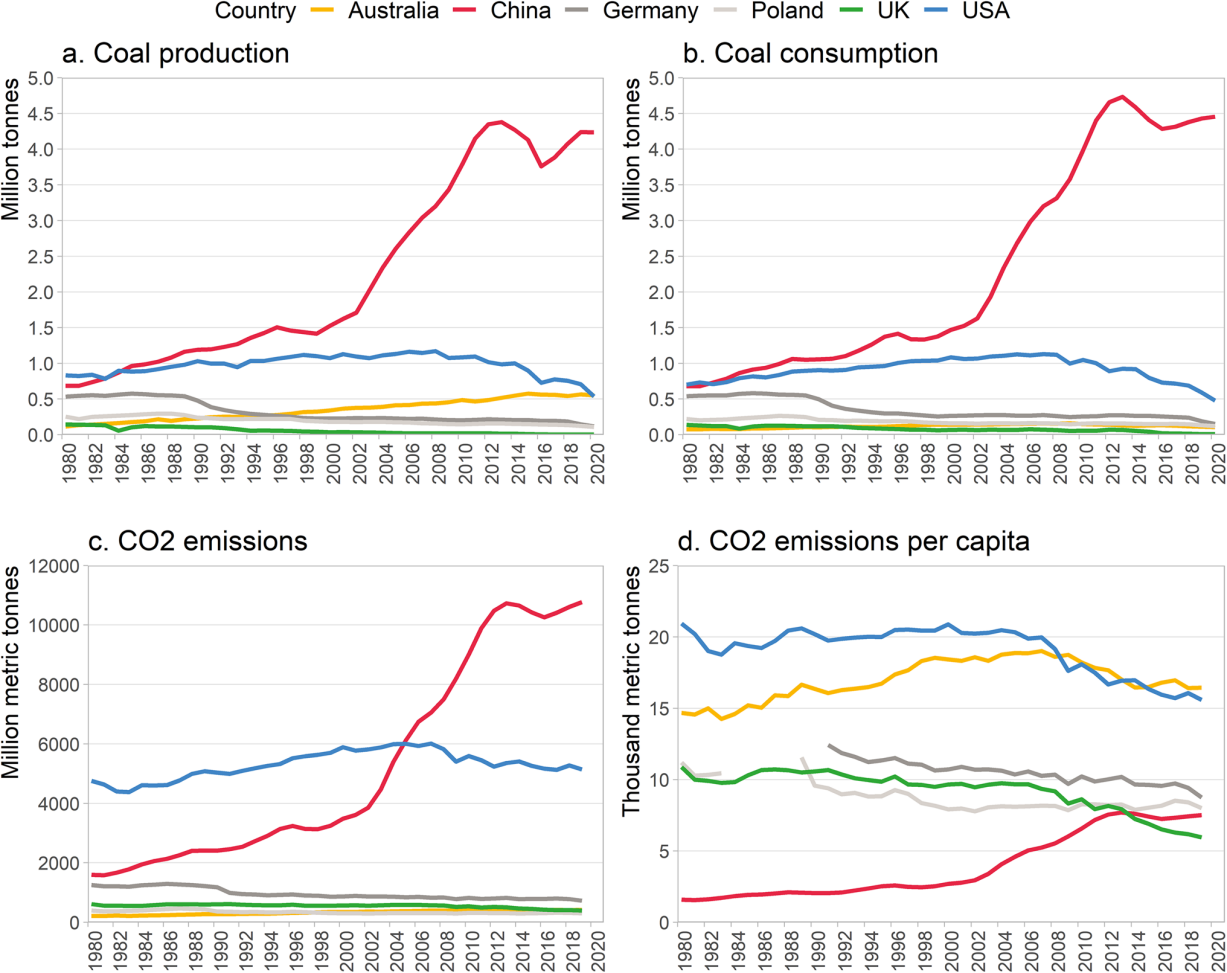
Coal industry and CO_2_ emissions in selected countries: **a** coal production, **b** coal consumption, **c** CO_2_ emissions, **d** CO_2_ emissions per capita. Between 1980 and 1990, data for Germany presented as Eastern and Western Germany combined. Population data is missing between 1980 and 1991 for Germany and between 1984 and 1988 for Poland

This review primarily draws on academic papers published in English that document coal transition experiences in Germany, the UK, the US, Poland, and Australia. These countries were chosen as their experiences have been well-documented in academic literature (Brauers and Oei 2020; Braunger and Walk 2022; Della Bosca and Gillespie 2018; Oei et al. 2020a, b; Diluiso et al. 2021). In examining their relevance for China, the paper has two limitations: the scale of the transition challenge and the importance of context-specific characteristics. Coal transition challenges in these countries have been significantly smaller than China’s. Figure 1 (a) compares coal production, (b) coal consumption, (c) total and (d) per capita CO_2_ emissions in the five countries. Although relatively low, the growth trend for China’s per capita CO_2_ emissions is concerning, having surpassed the UK’s in recent years.

Additionally, local socio-economic, cultural, and institutional conditions have shaped coal transition experiences elsewhere. Although the Chinese context bears significant differences in governance and political economy (Huang et al. 2021), it shares several drivers similar to those experienced in other regions. These include, among others, aging infrastructure, climate action, and poor air quality. Examining past experiences—successful and failed—may encourage China’s policymakers to draw on insights on governance and stakeholder engagement that could, in turn, assist planning to achieve ambitious climate commitments.

A targeted literature review was conducted using two databases, Scopus and Google Scholar. The review included academic peer-reviewed articles in the English language that discussed coal transitions in five selected countries. Due to the recent and emerging nature of coal transition policy in Australia, limited academic research was available. To fill this gap, Google searches were conducted that identified recently published reports on the topic of coal transitions. The search strategy included a combination of keywords specific to coal and country (e.g., ‘coal’ and ‘Germany’) and keywords associated with energy transitions (e.g., ‘transition’, ‘closure’, ‘phase out’, ‘exist’, ‘phase down’). More than 300 publications were identified.

In the second step, papers were included if they explicitly provided a summary of lessons or experiences from coal transitions in selected countries. All articles were reviewed and those considered irrelevant were removed. 72 papers were identified that matched our criteria. Nine papers provided lessons for more than one country. Over 50% of the publications were published in the last three years with their focus on Germany. For Australia, only one academic publication appeared in the search alongside four reports. See Table 1 for details on reviewed publications.

**Table 1:** Number of reviewed publications by year and country

Year	Number of reviewed publications*	Germany	Poland	The UK	The US	Australia
1996	1			1		
1997	1			1		
2002	1	1				
2005	1			1		
2010	1	1				
2014	1			1		
2015	3		2		1	
2016	2	2				
2017	4	3		1	1	
2018	8	2	3		2	2
2019	9	4			2	2
2020	15	7	2	5	1	1
2021	14	9	4	1		
2022	11	5	1	2	4	
Total	72	34	12	13	11	5

*Number of publications per year is not equal to the sum by country, as some papers focused on two or more countries

The final selection of selected papers is not exhaustive. The approach was not to provide a systematic review of coal transitions in these countries [see Diluiso et al. (2021) for a systematic review]. Instead, the aim was to target those papers that provided a breadth of experiences and approaches to coal transitions, with the overarching aim to draw relevance for China.

To analyze the content of selected publications, text was coded across four themes: governance, inclusive participation, focus beyond jobs, and alignment with existing policies. A systematic analysis was not possible, as selected publications varied in focus, aims, method, outcomes, and the scope of lessons.

The paper is organized as follows: we begin by discussing the idea of a just energy transition. The next section sets the problem context in China and introduces its coal transition challenge. Then, we examine the economic, social, and institutional background contexts within which the coal transitions of Germany, the UK, the US, Poland, and Australia were situated. Finally, relevant lessons from these experiences are collated to relate the idea of a just transition to China’s coal phase-down. The paper concludes with key findings.

## Arguing for a just transition

Scale and pace are critical factors to consider in energy transitions planning and policymaking. An accelerated top-down decarbonization process may pay poor attention to “process”, thereby compromising on equity, sustainability, procedural fairness and social legitimacy (Delina and Sovacool 2018). Calls are growing for a careful examination of the social, economic, and environmental impacts on regions likely to be affected by energy transition interventions, including both fossil fuel phase-out (Mayer 2022) and the growth in renewable energy development (Süsser and Kannen 2017; Avila 2018).

This has led to the (re)emergence of a “just transition” agenda (Carley and Konisky 2020). Coined in the 1970s as an idea fostered by labor union movements and environmental justice groups in North America, its focus was limited to worker rights, including jobs and livelihoods security. Over the last decade, it has achieved a renewed focus in the global push to address climate emergencies and plan energy transitions (Jenkins et al 2020). It was formally adopted in 2018 as part of the “Solidarity and Just Transitions Silesia Declaration”. The declaration called for “a just transition of the workforce and the creation of decent work and quality jobs [as critical to] … an effective and inclusive transition to low greenhouse gas emission and climate resilient development” (UNFCCC 2018, p. 3).

In broad terms, the idea of just energy transitions builds off discourses on energy, environmental and climate justice (Heffron and McCauley 2018), to include a focus on fair compensation for workers in coal-based sectors, reskilling and retraining, ecological restoration of lands affected by coal mining, and social revitalization of regions through infrastructure development, economic diversification, and cultural preservation (Jenkins et al. 2020). Additionally, it calls upon deep-seated readjustments: for example, the de-marginalization of groups and communities that have historically remained vulnerable to political and socio-economic abuse. In doing so, it offers the potential “to strengthen the remedial dimensions of the ‘protect, respect, and remedy’ framework that underpins the UN Guiding Principles on Business and Human Rights” (Bainton et al. 2021, p. 631).

These framings are reflected across the literature on just transitions which contains three key anchor points: a jobs-orientated approach led by unions that seek support from markets and states for impacted workers and regions; a socio-technical approach that considers the environmental gains resulting from a low-carbon transition; and a society-focused approach that positions just transition as the means to achieving the aspiration of a fair society, one that is equitable, thrives on a sound economy and preserves the region’s ecological integrity (Krawchenko and Gordon 2021). Despite positioning social justice at its core, so far, moves to operationalize the idea of a just transition retain an overwhelming focus on jobs, including reskilling and retraining (Heffron and McCauley 2022). Most policy support and financial interventions focus on this area, with little regard for the environmental restoration of affected sites or differentiated vulnerabilities across social groups. More attention is needed to integrate a focus on jobs with broader structural challenges and opportunities (Jenkins et al 2020).

Some progress toward integrating streams of social justice is evident; for example, the EU’s Just Transition Fund and the World Bank’s “Just Transition for All” initiative are pushing policymakers to consider people and communities at the “heart of the transition” (European Commission 2022a; World Bank 2022b). The three anchor points underlying energy justice contribute to its broadening scope through different forms of justice including: (a) distributional justice in energy systems to understand how and where impacts vary across different groups drawing on existing societal divisions across race, gender, caste, and class divides; (b) recognition justice to appreciate and recognize groups of interest that have been traditionally marginalized and explore where injustices emerge through the practices of cultural domination, non-recognition and disrespect; and (c) procedural justice that focuses on how societies and communities are included in the process of energy transition planning and decision-making such that outcomes are fair and equitable (McCauley et al. 2013, 2019). Forms of distributional, recognition, and procedural justice inform our analysis as we engage the idea of a “just transition”, consider coal transition experiences globally, and draw lessons for China.

## Challenge of a “just” coal transition in China

China is the world’s largest CO_2_ emitter. In 2021, its coal output of 4 billion tons contributed to more than 50% of the world's total. It has the world’s largest number of coal-fired power plants (> 1000 units) and operating coal mines (> 10,000). China is thus critical to global efforts to address climate change (IEA 2021).

In recent years, China has pursued policies of green and sustainable development underpinned by a political ideology of an “Ecological Civilization” (Dong et al. 2022, p. 1744). In 2020, China announced its commitment to achieve carbon neutrality by 2060 (NCSC 2021). The “deep” emission reductions thus require coal demand to drop to nearly zero by 2050. This would, in turn, necessitate a proactive, orderly closure of coal mines and coal-fired power plants (Duan et al. 2021). While Cui et al. (2021) offer potential coal phase-out roadmaps drawing on technical, environmental and economic assessments, an understanding of the social implications likely to result from such large-scale industrial transformation is missing.

China’s 14th five-year plan positions green development and social equality as central policy priorities (The State Council 2021). It stresses China’s ambition for an “Energy Revolution” suggesting large-scale reforms in the energy sector. While the Plan falls short of formally calling for a just transition, several priorities listed therein align with the principles of just transition discussed earlier. For example, it outlines actions for a more equitable regional development, including measures to improve employment opportunities and strengthen the national social security system. China’s policymakers have a timely opportunity to devise reforms that synergize the Plan’s priorities for well-being and social cohesion, and the nation’s broader climate and energy targets.

China has undertaken large-scale reforms in its coal industry in the past. To improve safety or prevent oversupply, industrial restructuring campaigns for “mine closure and production reduction” and “coal resources consolidation” took place between the late 1990s and 2010 (Cao 2017, p. 199). On the one hand, this resulted in more efficient and productive facilities run by state-owned enterprises (SOEs) owned by central, provincial or local administrations (Lin et al. 2020). On the other hand, by impacting employment numbers, salaries, and operating hours, this restructuring disrupted social cohesion. Current CO_2_ peaking and carbon neutrality programs prioritize guarding against economic and social risks. However, better guidance is needed to understand how such risks can be fully identified, prevented, and/or mitigated.^1^

**Fig. 2:**
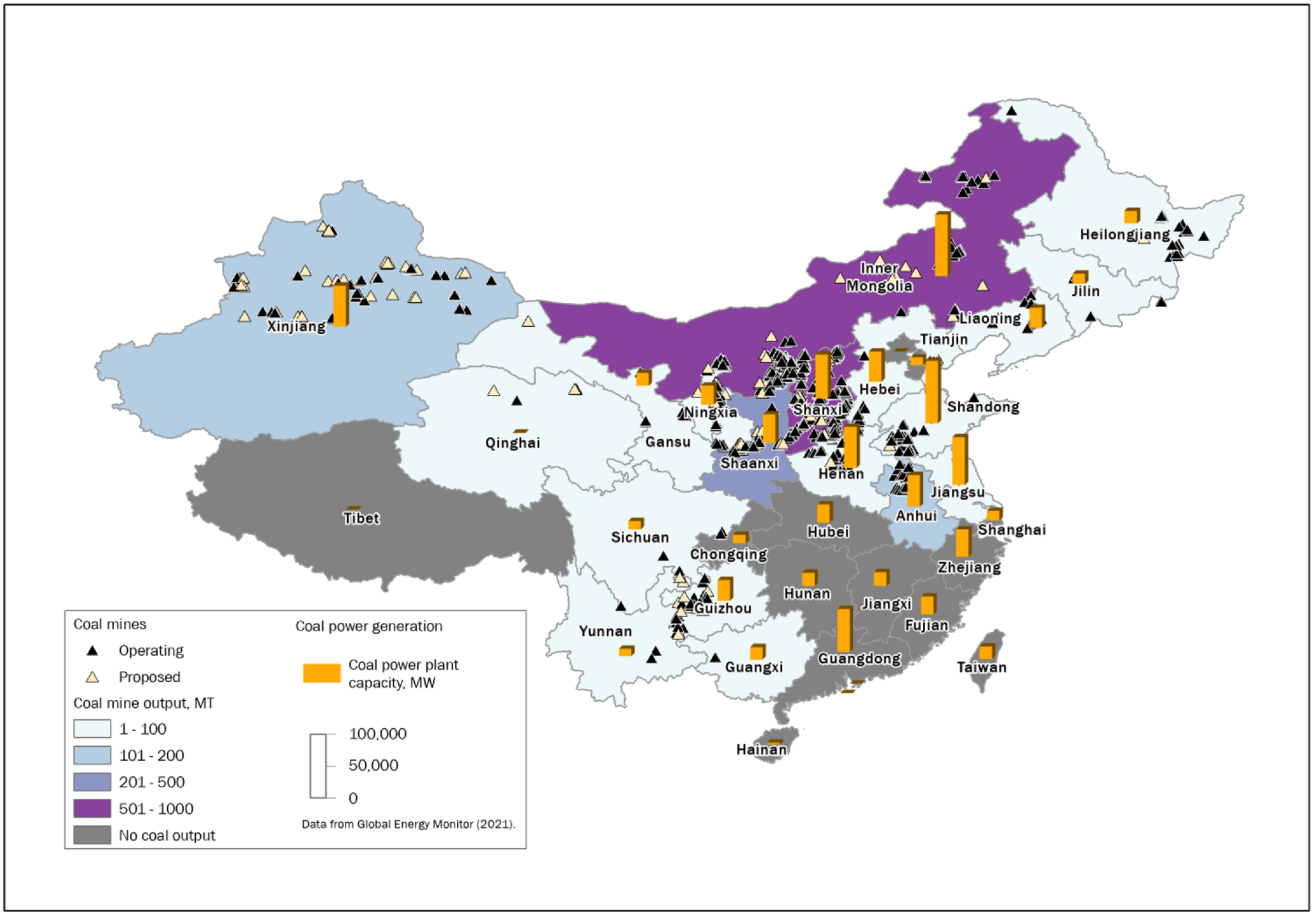
Coal mining and coal power generation in China. Source: Global Energy Tracker (2022)

In its China Climate and Development report, the World Bank (2022a) expressed support for a “just transition” in China through policy, financial support packages and place-based implementation. However, questions have been raised about the practicability of a just transition in China given China’s hierarchical and highly devolved, state-led political and economic system that is heavily reliant on coal (He et al. 2020; Hu 2020; Huang and Liu 2021; Wang and Lo 2022). While China’s regional and local governments have targets for coal phase-out, until recently, they were incentivized to develop the coal sector, creating a deep dependency throughout the country. Figure 2 demonstrates the spatial distribution of coal mine capacity and coal power generation across China’s provinces. Inner Mongolia and Shanxi stand out for their deep dependence on coal.

Past reforms suggest that SOEs are likely to have a major influence on China’s transition outcomes (Ohlendorf et al. 2022). SOEs will influence the creation of opportunities for retraining, protecting workers’ social welfare, and facilitating entrepreneurial models that diversify China’s economic base, and strengthen national stability. Drawing on first-hand evidence of a coal mine closure in Inner Mongolia, Wang and Lo (2022) highlight the need to better understand the role of the SOEs in furthering political and economic inequity across China. Their study found that while coal workers from state-owned coal mines were supported by job reallocation and retirement plans, workers from privately owned mines were laid-off without adequate compensation and job assistance. Private mine workers were mostly from rural areas and their rural *hukou*^2^ constrained their ability to seek jobs in urban settings when compared with those holding an urban *hukou*. The government too was hesitant in assisting rural workers as they were not considered its primary responsibility. Although *hukou* reforms were initiated by the National Development and Reform Commission (NDRC) in late 2021, the process of identifying and supporting vulnerable groups remains complex and politically divisive (Jaramillo 2022). Given the socio-economic heterogeneity both within and across China’s coal provinces, “just transition for whom?” is an important question in understanding and planning transition pathways.

The renewable energy sector is considered an alternative for countering employment losses in the fossil fuel sector, particularly in the Global South’s labor-intensive coal industry (Caiet al. 2014; Pai et al. 2020). Large solar energy projects in China’s remote areas are transforming coal-dependent regions into multi-energy systems (Pai et al. 2020). For these developments to generate equitable benefits, government policies would need to target China’s vulnerable and underrepresented groups. As noted elsewhere, lack of focus on the justice implications of shifts in energy systems can exacerbate gender disparity, rural–urban inequity, human rights abuse, and marginalization of vulnerable groups (Caiet al. 2014; Yenneti et al. 2016). This is particularly important for China where sensitivity to just and fair social and cultural outcomes of transitions are being over-shadowed by a near-exclusive focus on jobs: the loss of jobs in coal regions and the promise of “green jobs” from the rapidly growing renewable energy sector. Studies that consider wider social impacts on standards of living, patterns of inequity, access to affordable and clean energy, environmental restoration, and the role of small to medium enterprises in enabling diversified economies are sparse (He et al. 2020; PRI 2022).

## Global review of coal transition experiences: setting the context

Communities across the world have experienced a range of challenges in phasing out energy sources, including nuclear (Jarvis et al. 2022) and coal (Oei et al. 2020a, b; Abraham 2017). Coal transition experiences have been widely studied (Diluiso et al. 2021) and offer important insights for contemporary energy transition policymaking.

This section examines five national experiences with coal phase-out. They encompass both success stories and challenges that remain to be addressed. The rationale to bring together these lessons is two-fold: first, to urge countries, such as China (and other coal-dependent regions including India, Indonesia, Vietnam, and South Africa) to learn from others’ failures in applying just transition principles in their respective journeys toward fossil fuel independence. Secondly, the diversity in experiences enables a broad-based understanding of transition processes, while reiterating the significance of local and national contexts, thereby cautioning against a “one-size-fits-all” approach to energy transitions.

### Germany

In the late 1930s–early 40s, Germany’s coal industry employed over 600,000 people directly and indirectly. Much of the coal production was concentrated in two areas: Ruhr and Saarland. At their peak, these two regions produced over 130 million tons annually, placing them 8th in total coal production in current times, above Kazakhstan, Poland, Turkey, and Colombia (World Energy and Climate Statistics 2022). Despite political and demographic differences, both regions shared strong socio-economic identities shaped by the presence of a steady coal economy over nearly two centuries. As Europe’s most important coalfield at the time, the Ruhr region was considered a national asset and was one of the most densely populated regions in Europe (Arora and Schroeder 2022; Goch 2002).

Since the 1960s, the German coal footprint has been on a steady decline, driven by cheaper overseas oil and coal. By the 1990s, under 100,000 people were employed in the sector, falling further to approximately 3000 in 2018 when the Ruhr’s last hard coal mines permanently closed. By comparison, the Saarland region’s coal operations closed in 2012. While Germany still operates lignite mines, production has declined steadily, and plans have been announced to end lignite mining by 2038 (Oei et al. 2020a, b). The war in Ukraine has led to serious disruptions to the German energy supply, prompting both hard coal and lignite power stations to restart operations at the time of writing (Bryce 2022; Dezem 2022). This has led to an increase in coal imports over the short to medium term. Early projections proposed German coal imports in 2022 to grow by more than 10% from the 2021 level (Kinkartz 2022).

Notwithstanding these recent developments, coal decline in Germany occurred independent of other nationally significant energy-related developments over the last several decades. These included the nuclear energy phase-out in 2011 and the growth in the uptake of renewable energy (mainly wind and solar) driven by technological advances that improved their affordability and accessibility (Renn and Marshall 2016). All along, Germany maintained its long-standing support for the coal industry through generous subsidies. The *Energiewende*-fuelled expansion of solar and wind energy was firmly embedded within a publicly supported policy stance to phase out nuclear energy (Renn and Marshall 2016). This high-paced uptake of renewable energy did little to expedite Germany’s coal phase-out. Decarbonization merely remained an aspirational target until the introduction of specific EU legislation in the mid-2010s.

The German coal sector has traditionally maintained strong political clout alongside a powerful trade union. This was most evident on two occasions: (i) the rejection of a “climate contribution” that would have allowed the timely closure of older inefficient coal plants (Oei et al. 2020a, b), (ii) decisions around the timing of the German coal phase-out and resistance to the adoption of EU air quality regulations. The unions pushed for a “gradual” coal phase-out alongside demands for benefits and support packages for affected workers (Steckel and Jakob 2021). High political capital of the coal regime systematically fueled regional and national concerns over job losses, energy security, and import dependence, thereby allowing Germany to outlast its European counterparts in sustaining the coal sector (David and Gross 2019).

### Poland

Poland is the EU’s largest hard coal producer and the second-largest lignite producer. It produces over 110 million tons of coal annually from over 20 active coal mines, concentrated mostly in the Upper and Lower Silesia regions (Global Energy Monitor 2022). Poland’s current coal production is more than half its peak production in 1987. Coal constitutes over 70% of Poland’s electricity generation and over 40% of the total energy supply. The lack of access to other energy sources makes Poland one of the most coal-dependent economies in Europe (Brauers and Oei 2020). The industry employs nearly 85,000 miners, mostly in the province of Silesia.

The coal sector was the main driver of Poland’s regional development through the nineteenth century. During Soviet rule (1945–1989), the coal industry was nationalized and centralized. Systemic inefficiencies and an excessive labor force resulted in a dysfunctional sector by the mid-1980s, defined by poor productivity and unprofitability (Kowalska 2015). Fueled by a free market, the following three decades saw Poland’s coal industry undergo widespread social and economic transformation. Productivity and profitability improved as 42 of the least profitable mines were shut down between 1990 and 2006 (Kowalska 2015). This caused significant decline in coal production and widespread job losses (from 400,000 miners in 1990 to just over 80,000 in 2019).

Since the state reinstated control over the industry, demand for, and use of, coal has remained strong in Poland despite the sector’s low profitability. Concerns over energy security and independence, and the presence of strong trade unions have shaped broad support for coal use (Brauers and Oei 2020). Consequently, calls for industrial transformation have sought policy assistance for job creation, and region-specific funding to address poverty and social exclusion. To better align with recent shifts in the EU climate and energy policies and growing civil society demand for coal phase-out, the Polish government reached a socio-economic agreement with the miners’ unions in 2021, to close all coal mines by 2049. The government has also introduced Just Transition Plans, recognizing the need for fairness and equity in mapping transition impacts on affected regions and communities (National Reform Programme 2022). At the time of writing, the war in Europe has affected Poland’s energy outlook for late 2022–early 2023. Embargo on Russian imports has exposed domestic vulnerability to global energy shifts. Reports suggest the burning of household trash as one of the many substitutes for heating purposes, further diminishing air quality in some of Europe’s most polluted cities (Martewicz and Skolimowski 2022).

### United Kingdom

From the mid-to-late-twentieth century, the UK’s coal mining industry underwent widespread labor, industrial and socio-economic transitions. The once-powerful coal unions fought against mainstream political establishments to lobby against coal mine closures. The 1980s marked the beginning of the sector’s irreversible and sharp decline, as miners opposed the Thatcher government’s policies to liberalize the national energy landscape (Beatty and Fothergill 1996). Within ten years (1984–1994), the number of wage earners fell from over 180,000 to 10,000 as collieries closed at an unprecedented rate (Glyn and Machin 1997). Overall, jobs in the coal economy dropped from 200,000 in 1985 to 7000 in 2005 (Johnstone and Hielscher 2017).

While the decline in coal mining did not affect the UK’s coal consumption, there were clear shifts evident in the government’s position on coal (Isoaho and Markard 2020). By the mid-1990s, growing political pressure from environmental groups had led to climate change and “green energy” becoming defining policy tools that dismantled the political economy of coal in the UK (Johnstone and Hielscher 2017). From a near-equal share between coal, gas, and nuclear in electricity production, by 2020, the share of coal was only 2%, with natural gas and renewables supplying the bulk, and only four coal-fired power stations operating at the turn of this decade (Stognief et al. 2022).

The UK’s coal transition experience suggests mixed results. Several policy and financial support interventions were implemented to manage the social and economic costs of a rapid coal phase-out. Public sector schemes aimed toward economic regeneration of coal mining regions successfully harnessed the UK’s location within the broader European regulatory environment (up until January 2020). A case in point is the EU’s RECHAR program, which focused on assisted regeneration of coalfields (Bennett et al. 2000). As a response to, and supported by the program, the UK established a Coalfields Task Force to drive targeted interventions to improve the social and economic living conditions in ex-coal mining regions (Beatty and Fothergill 1996).

While several of these regions were able to prevent long-term social and economic decline. labor market struggles remain commonplace in other regions. This is evident by below-par employment rates, over-reliance on manual jobs, higher-than-national-average self-reported ill-health, and a significant percentage of the population on disability support. Many regions affected by the mines closing remain poor, with limited well-paying livelihood opportunities, and unemployment rates higher than the national average, particularly in the aftermath of COVID-19 (Beatty and Fothergill 2020).

### United States

The US has had a strong and long relationship with coal. 22 of its 50 states are part of the three national “coal regions”. The most socially and historically significant of these is the Appalachian coal belt, encompassing eight states, and responsible for over 25% of the entire US coal production. West Virginia, the region’s largest coal-producing state and the second largest in the United States maintains deep ties with coal. In 2017, coal mining and coal-fired power generation supported nearly 40,000 people in the state, and contributed to approximately 17% of the state’s GDP (Snyder 2018).

Although coal provided over half of the national total power consumed between 1961 and 2008, important shifts in the coal landscape are evident (Wishart 2019). No new coal-fired power station has been built in the last decade, coal production is at a 40-year low, and the share of coal-fired electricity has reduced by 27 percentage points between 2003 and 2019 (Lu and Nemet 2022). From 1 million people working in coal mining in 1920, employment numbers have dropped consistently to approximately 42,000 in 2020. Despite these shifts, 26 counties across ten US states remain dependent on the coal sector for economic opportunities, jobs, and local government-supported social services (Morris et al. 2019).

Two key drivers help explain coal’s decline in the US: first, the emergence of cleaner, cheaper alternatives (shale gas, solar, and wind) have curtailed domestic coal use while export prospects remain weak due to regulatory constraints and uncertainty in the Asian markets (Lu and Nemet 2022). Second, anti-coal calls from pro-climate groups have forced power utilities and financiers to divest from coal to maintain a “progressive” and responsible public image (Kang 2016).

The decline is noteworthy given the US has one of the world’s most influential coal lobbies. Institutional and political actors on either side of the fossil fuel narrative have politicized America’s energy trajectory on issues of affordability, security, employment, and climate responsibility (Hermwille and Sanderink 2019; García-Muros et al 2022). For decades, the coal industry has financed political campaigns, wielding, in turn, enormous power to influence policymaking both at the state and federal levels. However, there is evidence of a weakening coal lobby (Sicotte et al. 2022) as the industry is projected to lose approximately 12,000 jobs annually over the present decade (Mayer 2022).

The potential loss of employment and the impacts it would have on the social and economic fabric of coal-dependent regions were critical pillars of the Green New Deal proposed in 2019 (Galvin and Healy 2020). Several ex-coal regions across the US grapple with higher-than-national-average poverty rates, high levels of socio-economic decline and environmental degradation (Snyder 2018). Consequently, there have been growing calls for policies building on just transition principles to target regional development across America, particularly in the Appalachian regions (Carley et al. 2018; Sicotte et al. 2022).

### Australia

With an output of over 500 million tons per year, Australia is the world’s second-largest coal exporter, after Indonesia. Exports include nearly all coking coal and around 70% of thermal coal produced locally. Domestic reliance on coal also remains significant, as it accounts for more than 50% of the total power generation. Currently, there are nearly 100 operating coal mines in Australia with most black coal mines located in Queensland and New South Wales (NSW) while brown coal is present further south in Victoria. Approximately 40,000 people are employed in coal mining, accounting for one-fifth of all mining employment (AISC 2021).

Australia’s coal sector has played an important role in supporting both national and state economies. Despite ongoing growth in coal mining and exports, domestic coal consumption is under heavy scrutiny from investors and the wider civil society. This has resulted in declining coal consumption in the last decade, and the closure of several coal-fired power plants. Nine coal-fired generators have retired since 2012, while others face imminent closures, including Australia’s largest (Eraring in NSW) scheduled to close by 2032.

One of the most widely discussed thermal power plant closures in Australia’s recent history is the Hazelwood plant near Melbourne. At the time of closure in 2017, it was Australia’s oldest, dirtiest, and largest power plant by capacity (Burke et al. 2019; Wiseman et al. 2020). Multiple concerns led to the plant’s closure: the introduction of carbon pricing leading to marginal increase in plant operating costs, a bushfire incident causing an uninterrupted fire at the mine leading to community concerns over public health and the environment, the state government’s increased rate for coal royalties, and the need to upgrade and repair infrastructure to meet workplace health and safety regulations. The plant’s decommissioning entails considerable investments, with costs of over A$700 million and a 30-year timeframe for site rehabilitation (Jotzo et al. 2018).

The Hazelwood plant closure has been a “mixed bag” of experiences. Significant financial packages from the state (A$266 million) and federal governments (A$43 million) supported local infrastructure development, economic diversification, and job creation (Jotzo et al. 2018). Yet, concerns have been raised over the timing and efficacy of consultation processes between companies, worker unions, governments, and community stakeholders (Wiseman et al. 2020) (Table 2).

**Table 2:** Summary of international experiences with coal phase-out and relevance for China

Regions	Drivers	Governance	Successes	Failures	Relevance for China
Germany (transition from 1982)	Continuous planned and controlled coal decline	Coal Commission with a multi-level, multi-sector focus on identifying and managing transition impacts. Complemented by substantive financial commitment	Promotion of new identities and economic opportunities in coal regions to offer sustainable and long-term alternatives	Policy interventions were not able to prevent externalities of structural change, including negative impacts on the labor market, demographic decline and outmigration	The significance of polycentric and multi-level governance; institutional certainty of funding and a set timeline
Poland (transition from 1987)	Shift to market economy and associated sectoral shifts, high costs of coal production, poor economic viability (high production costs, poor labor productivity)	Financial compensation for the coal industry, coal regions and workers; training programs	Support from municipalities	Negative experiences of insufficient support, causing structural breakdowns. Widespread fear and institutional distrust leading to strong opposition against upcoming coal transitions; lack of focus on environmental remediation in coal regions	Planned transition with support from trade unions; localized place-based planning
The US (transition from 1998)	Competition from alternative energy sources (shale gas, renewable energy); anti-coal calls from pro-climate groups	State-driven attention to just and people-centered transition planning	Focus on economic diversification led by local champions and rooted in community ties	Lack of attention to environmental governance and land rehabilitation. High levels of socio-economic decline persists in ex-coal regions	The role of local champions; focus on job creation is insufficient without considering quality of jobs created
The UK (transition from 1981)	Coal decline in primary consumption led to an abrupt collapse of coal production, poor economics of coal, aging infrastructure and the emergence of climate action	Stringent regulation on coal industry	Local approach to and community-led design of regeneration interventions	Top-down implementation of large funds failed to address vulnerabilities of those most at-risk, leading to further marginalization and inequity	Programs are likely to be successful when responsive to community needs
Australia (transition from 2012)	Increased plant operating costs, bushfire accident leading to community concerns over public health and the environment, state government’s demand for increased coal royalties, and aging infrastructure	State (province)-based decision-making	Focus on local infrastructure development, and economic diversification	Insufficient time invested in stakeholder consultation	Significance of aligning transitions plans with local priorities; instability in national climate and energy policymaking is disruptive to streamlined action

## Discussion and key learnings for China’s coal phase-out

Coal mining and coal-fired power plant phase-out experiences of the five regions presented above collectively span more than five decades. They have each been shaped by distinct national and local social, economic, political, and ecological factors. While different to China, this section draws out lessons from elsewhere and considers their significance for China given the idea of a just transition (Zhang and Chen 2022).

China’s development ambition—set through its 5-year plans—suggests an intent to operationalize key values underlying a Chinese “just transition”, namely social cohesion, rural–urban revitalization, and ecological civilization (The State Council of the People's Republic of China 2021). Past domestic and international experiences with energy transitions provide China lessons to plan pathways that proactively engage with these values and principles. Building on the successes but also importantly, the failures, we identify four policy pillars that provide useful pointers for energy policymaking in China over the next several decades.

**Fig. 3:**
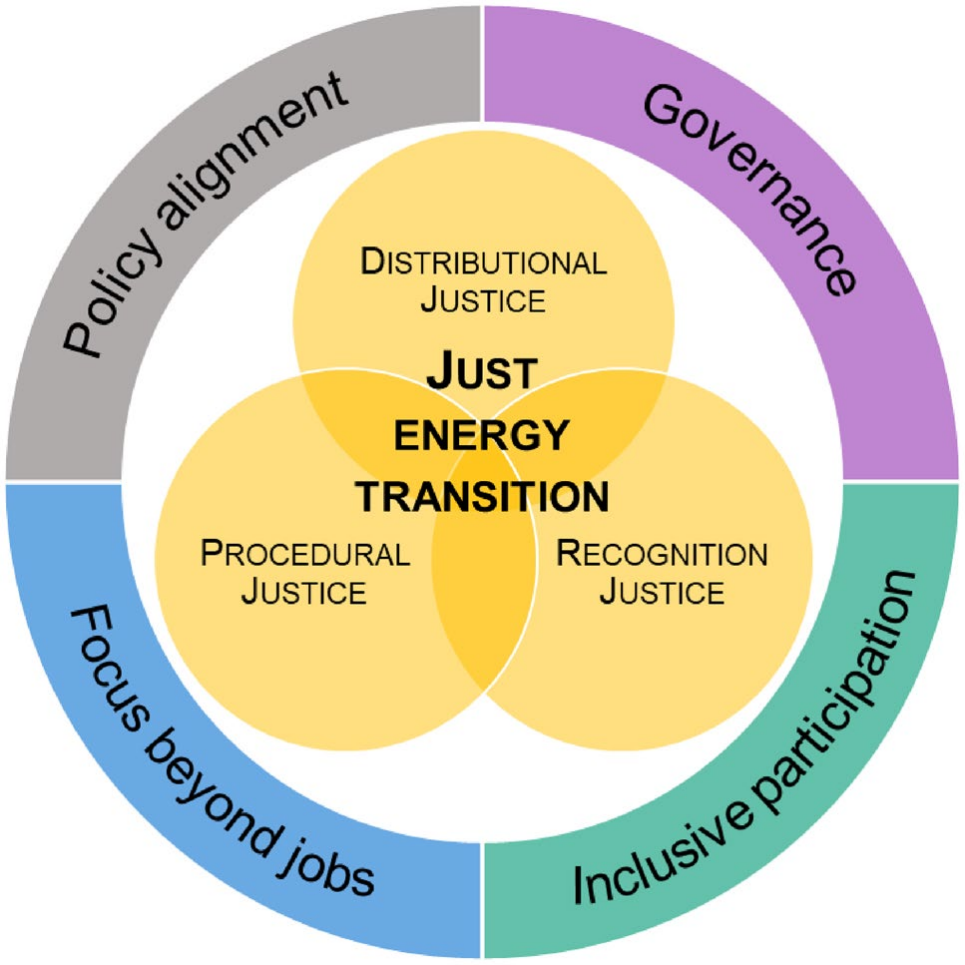
Guiding Framework for China’s Just Transition

Each of these pillars advances the three tenets of distributional, recognition and procedural justice. The pillars operate in a continuum; treating them in isolation is likely to be counter-productive to the long-term transitions agenda. Progressive interventions in one policy area influence and shape outcomes in another. Consider, for example, communities at risk of social and economic marginalization as a result of coal phase-out. Identifying these groups by engaging across public, private, and civil society actors for assistance with place-based support allows multiple co-benefits that collectively advance the prospect of mobilizing just transition principles (Fig. 3).

### Governance

Governance was a critical factor in all five countries. Energy transitions are deeply complex and occur within institutional and politico-social structures (Berkhout et al. 2012). Understanding these background complexities and structures, while designing open, accountable, and tactical governance frameworks can help in the planning, management and administration of long-term transitions (Loorbach et al. 2008). Polycentric governance can support regional and national governments to commit resources proportional to the scale of the challenge (Oie et al. 2020a, b; Furnaro et al. 2021). Good governance can also offer some temporal certainty to transition processes and those affected by it. A realistic timeline conducive to a progressive transition has allowed some regions and communities to address proactively socio-economic restructuring and environmental rehabilitation (Arora and Schroeder 2022).

The German experience is a case in point. To decarbonize its electricity fleet and identify a strategy to meet the country’s broader energy transition goals, the federal government set up the “Commission on Growth, Structural Change and Employment”, known as the Coal Commission (Furnaro et al. 2021). Its scope was two-fold: developing strategies to ensure economic development, social compatibility and cohesion, and climate protection, and identifying fiscal measures to support large-scale structural change in coal regions and communities. Through its multi-stakeholder consultations (see below), the Commission highlighted the need for different levels of governments to commit resources through a variety of structural programs. With support from local governments and large federal financial interventions, programs targeted specific geographical areas undergoing transformation. One such program in the Ruhr region focused on improving connectivity and transportation infrastructure. This had multiple positive effects: it allowed mobility between key cities and opened former mining regions to new research and entrepreneurial opportunities. This helped demonstrate the region’s collective desire to host new initiatives and transform its image from a conservative mining region to one that was culturally and ecologically progressive (Schepelmann et al. 2016). Stakeholders in the German transition process were able to harness—and protect—existing regional identities firstly, by re-creating “cultural” value based on existing assets (e.g., old infrastructure and mine sites) and second, by embracing a (re)development plan built around innovation and research, ecological restructuring, and a tertiary economy (Goch 2002).

Similar efforts were undertaken with varied success in Poland and the UK. In Poland, a commission for social dialog was initiated, thereby inviting input from municipal, regional, national and European actors (Hielscher et al. 2022). The commission is considered partly successful in developing a just energy transition plan containing regional strategies supported by targeted funds to tackle issues concerning the environment, social capital, and transport.

In the UK, the Coalfield Regeneration Trust was a key non-government entity set up to monitor and seek accountability from government structural programs (Coalfield Regeneration Review Board 2010). Besides being the UK’s longest-running, largest financial intervention, receiving GBP300 million in public funding over 20 years, the program design was responsive to community needs and vision and was well received by affected communities.

For China’s sustained transition out of coal, an open, accountable and tactical governance framework is critical for three reasons (European Commission 2020): (i) to draw together resources from across multiple administration levels to support the heterogeneity across coal-dependent regions and recognize the diversity of needs at the grassroots level, (ii) to provide temporal certainty of planned closure to affected communities, and (iii) to facilitate co-ownership and co-management of restructuring programs, thereby building capacity across urban and rural, large and small industrial regions across China.

A governance framework that operates across scale is a critical enabler for just transition principles to find support in China. Designing such a framework that respects and maintains alignment with broader institutional decision-making sensibilities will improve capacity within China’s provincial and local authorities to navigate change brought about by energy and climate transitions (World Bank 2022a). This would, in turn, enable greater appreciation for dependencies across China’s coal supply chains that extend beyond jurisdictional boundaries. Coordinated inter-provincial institutions that consult with key stakeholders along these supply chains are likely to support greater social stability and cohesion as China plans systemic transformations.

### Inclusive engagement with a focus on process

The idea of a just transition stems from energy, climate, and environmental justice discourses that bring “stakeholders of all types to the transition process … [making it] inclusive” across space and time (Heffron 2021). Past efforts in managing the impacts of coal phase-out may be attributed in large part to the process followed in achieving procedural, recognition and distributional justice at the local, regional and/or national scale. Programs that recognized, and acted upon, the differentiated vulnerability of communities, regions and stakeholder groups were successful on at least two fronts: first, they were able to effectively engage, and extend government financial support toward regions and communities most in need. Second, and consequently, there was greater uptake of co-designed transition plans and programs by the local communities. For example, the German Coal Commission comprised 31 representatives from key affected regions, trade unions, industry, environmental associations, and the federal parliament.^3^ An inclusive multi-party approach to negotiating and co-developing a “transition agenda” helped embed it within a dialog-driven model to create jobs, revitalize infrastructure, and compensate affected households and communities (Arora and Schroeder 2022). Although the Commission’s recommendations are not legally binding, its focus on processes of inclusion will likely provide broad-based support for financially sound and socially just decision-making to achieve Germany’s planned phase-out by 2038 (Reitzenstein and Popp 2019).

In the UK, inclusive programs found more success. The “Regional Growth Fund” initiative (2010–2017) provided financial assistance to small enterprises (Ward 2016). A local approach to regional regeneration led to its success, particularly in engaging with the private sector. By 2015, it had created or safeguarded over 140,000 jobs. By contrast, the British Coal Enterprise—the British Coal Company’s “job-creation arm” was less successful. As a public sector initiative aimed toward economic regeneration of coal mining regions, its top-down implementation lacked a consultative process to identify miners’ specific context and employment needs. The new jobs created were not targeted toward miners. Most new jobs were taken up by community members with higher education credentials. This disadvantaged the miners and exacerbated distributional injustices in several coal regions across the UK (Beatty and Fothergill 2020).

Studies indicate that public engagement for major project development is underdeveloped in China (Zhang et al. 2022). Inclusive engagement is difficult to implement at provincial and local levels (Wang et al. 2021), and across the private sector (World Bank 2022a). While useful, lessons from elsewhere will require recognition of, and adaptation to, the Chinese context. With a centralized institutional structure, weak worker unions with limited bargaining powers, powerful national and regional governments and limited labor rights at the local level, an inclusive, dialog-driven multi-stakeholder process of consultation will be challenging in China (Huang and Liu 2021) and require innovative measures. This would entail “governmental institutions … play[ing] a vital role in communicating, educating, consulting and even collaborating with residents rather than just informing about … decisions” (Li et al. 2020, p. 9). While command-and-control interventions have been the norm, China’s energy transitions planning is showing signs of adopting market instruments and public participation; for example, a shift from “informing” to “consulting and collaborating” is evident in the country across forest restoration and climate governance (Long et al. 2018; Huang et al 2020). For just transition principles to find application on the ground in China, an important next step is to develop and test inclusive approaches to stakeholder mapping and consultation. This can promote engagement with private and civil society players within China who bring both interest and influence (World Bank 2022a).

### Focus beyond jobs

Past global coal phase-out experiences tend not to extend beyond jobs. Transition-related policymaking primarily addresses local economic impacts even though the social and cultural impacts and environmental disintegration of local landscapes are crucial to mobilizing the core principles of a just transition (Wilgosh et al. 2022). This narrow focus is akin to Fraser’s (1995) distinction between “affirmative” action on injustices vis-à-vis “transformative” action. The latter calls for “[addressing] inequitable outcomes precisely by restructuring the underlying generative framework … [including] restructuring of relations of production and recognition” (Fraser 1995, pp. 81–7). A rigid and traditional economics-driven, job-orientated approach to transition planning has been unable to address underlying structures that have aggravated environmental and social neglect in coal communities across the UK and Appalachia in the US.

Despite financial government support packages and structural reforms, several former coal mining regions report a poor quality of life. Simply presenting options for potential employment to affected workers without considering their interests, skills and societal expectations has not addressed economic inactivity among ex-coal mine workers in the UK (Murray et al. 2005). Similarly in the late 1970s–early 1980s period in the US, as federal power undermined organized unions and miners lost negotiating power, they experienced “spatial dispossession” (Smith 2015) leading to a long-term decline in their social, economic and physical well-being. This was exacerbated by the closure of public services in those same locales (e.g., rural post offices, schools, and common lands) and institutional neglect of ecological rehabilitation.

Gradual policy shifts away from a unidimensional jobs-focused approach are already underway in parts of the US and other places, such as Indonesia and South Africa. The release of Just Energy Transition Partnerships in both countries, for example, entail support for infrastructure development, entrepreneurial growth, protection of at-risk and vulnerable population groups, and cooperation and involvement of all actors along national and regional coal-dependent value chains (Colorado Department of Labor and Employment 2020; European Commission 2022b).

Drawing on these, it is worthwhile deconstructing China’s political and economic structures to advance a socially embedded understanding of structural barriers that exacerbate distributional and recognition injustices of coal phase-out. This may help in at least three important ways:

(i) *Recognizing cultural ties*: it would encourage decision-makers to recognize place attachments, including the cultural significance of individual and collective identities shaped by the presence of the coal industry.
(ii) *Mainstreaming gender*: overall, gender representation in mainstream Chinese development remains low and female employment in China’s coal regions is lower than the national average. This is evident in the declining trend of female workforce participation due largely to large-scale industrial restructuring; early retirement age for women; growing income disparities between men and women; and large gender-based disparities in political representation. Gender-sensitive transitions policies (Braunger and Walk 2022) would help improve women’s access to high-quality jobs, services that facilitate women’s participation in the workforce, and consultation processes that recognize—and work to address—underlying social and structural factors resulting in gender-based power asymmetries (e.g., cultural norms, poor recognition of unpaid work).
(iii) *Strengthening China’s long-term ecological integrity*: adequate government support for mined land rehabilitation (World Bank 2022a), complemented by regulatory penalties for lack of action on environmental restoration can address the underlying triggers of social conflict and economic decline. Several land rehabilitation interventions in China have been implemented successfully in the past (Zhao et al 2020; Yu et al 2022). Yet, the scale of rehabilitation funds, technologies and policies needed over the next few decades will be unprecedented in China’s mining history. This would require energy transitions planning, including the design of national and provincial coal phase-out strategies to entail a far more coordinated focus on the environmental integrity of affected landscapes.

### Alignment with existing policies

Co-benefits can occur when energy transition policy goals align with national priorities (He et al. 2020; Jennings et al. 2020; Department of Economic and Social Affairs, United Nations 2021; International Climate Initiative 2019). The German Coal Commission is a case in point. Its mandate was to negotiate transition pathways among different stakeholders and initiate a long-term plan to achieve Germany’s broader energy and climate ambitions. Despite challenges in implementation, the commission fostered a multi-pronged approach to achieving concurrent objectives, with policies remaining iterative and reflecting local, EU-wide, and global developments. It simultaneously considered social and economic impacts of coal phase-out on affected communities, implications for Germany’s energy security, and an orderly path for triennial monitoring of progress toward climate targets (The German Coal Commission 2019).

Unlike Germany, misalignment was evident in the case of Australia’s Hazelwood power plant closure. Despite significant state and national financial support, the lack of a national roadmap on coal mine and coal-fired power plants closure, unstable energy and climate policy, and poor institutional capacity as a function of the wider political turnover constrained good practice (Jotzo et al. 2018). As an example, the Australian Energy Market Commission (AEMC) requires electricity companies to provide a minimum of 3.5 years’ notice of upcoming closures. The prescribed notice period does not allow the sector sufficient time for inclusive and dialog-driven consultation processes in the development of new energy projects or address social and economic impacts from project closures. The Australian Government’s failure to recognize the climate emergency meant ad hoc interventions across the country without a coherent long-term coal phase-out strategy (Jotzo et al. 2018).

These lessons are relevant for China where five-year plans guiding national priorities offer a credible anchor to develop and implement energy transition pathways. Embedding the principles of a just transition within the broader national and regional development priorities can shape positive outcomes on multiple policy fronts (World Bank 2022b). Currently, gaps between “the central government’s vision and local governments’ practice” have been noted (Zhou et al. 2022, p. 1764). Re-alignment of national goals will further China’s energy transitions planning on at least two fronts: (i) bridging these gaps by recognizing at-risk groups along coal value chains and implementing place-based approaches to development; and (ii) designing interventions to address structural barriers that prevent the realization of financial, industrial, and societal co-benefits from green transitions.

Central planning and development agencies such as China’s NDRC will play an important role in building provincial and local government capacities to achieve the national priorities of social cohesion, and strong links between urban and rural development. This may require, for example, identifying where and how the current design and implementation of regulatory instruments (e.g., the SSRA) is insufficient in addressing the social impacts of energy transitions. Exploring application of the SSRA in identifying and mitigating social stability risks associated with coal phase-out and green energy development is one example of a potential synergy between existing regulatory instruments and the institutional capacity to mobilize just transition principles.

## Conclusion

China’s energy transition has a central role in the global effort toward decarbonization. The enormity of China’s reliance on coal calls for innovation in policy design and dynamic engagement with both state-owned and private sector stakeholders.

Energy transitions are simultaneously technological, economic, financial, political, and social. A unidimensional technocratic approach to energy transitions that “favor[s] economic considerations has to contend with conflicts arising out of a clash of objectives, values and visions of the social world” (Doering 2014, p. 1015). China’s policymakers are acutely aware of these sensitivities and are working toward transition outcomes that do not create social and institutional tensions that disrupt social and political stability.

There is little social science research that provides transition guidance to China’s policymakers and practitioners. This review paper aimed to address this gap by reflecting on developments in western countries in relation to the drivers, successes, and failures that underlined large-scale coal phase-outs over the last several decades. Notwithstanding the scale and complexity of China’s challenge and its unique formal, centralized style of decision-making, experiences from Germany, the UK, the US, Poland, and Australia offer insights for China’s approach to a coal phase-out. Four policy pillars are highlighted: governance, inclusive engagement, a transformative agenda that extends beyond jobs, and alignment in national policies.

A governance framework that supports strong and accountable institutions can support spatial, financial, and temporal certainty in transition plans. However, in China’s context of “a highly fragmented yet strongly authoritative state apparatus” (Cai and Aoyama 2018, p. 73), polycentric governance arrangements such as those observed in Germany may be difficult to achieve in the short term. Instead, a phased introduction of institutional autonomy and adaptability (Carlisle and Gruby 2019), evident in some of China’s past projects, may be appropriate (Downing et al. 2021).

The social stability quotient of China’s transition policies will be driven by the nature of engagement among diverse stakeholder groups. Evidence suggests that an inclusive, dialog-orientated approach to co-designing transition plans leads to fairer processes and outcomes. A holistic perspective that extends beyond a focus on jobs and addresses structural social and cultural barriers offers a credible pathway to longer-term transformation. Finally, embedding the idea of just transitions within development policy priorities can foster co-benefits, thereby contributing to greater policy ownership and uptake.

The lessons presented here are not exhaustive. Energy transition pathways are complex, context-dependent, and highly contested, with questions of justice at their core. For a country as large and heterogeneous as China, multiple ideas of “justice” in energy transitions are likely to emerge over the next few decades. The past global experiences of coal transitions examined here offer China guidance in navigating toward its vision of a high-quality, “common prosperity” growth model.

## Footnotes

1 While outside the scope of the present paper, one avenue is to explore the application of existing regulatory processes, for example, China’s Social Stability Risk Assessment (SSRA), in predicting, mapping, and responding to potential social risks from coal transitions (Peng et al. 2021; Zhang et al. 2022).

2 The *hukou* is a Chinese government-issued household registration system. China has two forms of *hukou*: urban and rural. The urban *hukou* is important for accessing employment, university education, and real estate in the urban areas. Rural *hukou* is often held by farmers in rural areas with relatively poor living conditions.

3 Included parliamentarians did not have voting rights. This ensured the Commission’s motivations and recommendations remained apolitical.

## References

[CR1] Abraham J (2017). Just transitions for the miners: labor environmentalism in the Ruhr and Appalachian coalfields. New Polit Sci.

[CR2] Arora A, Schroeder H (2022). How to avoid unjust energy transitions: insights from the Ruhr region. Energy, Sustain Soc.

[CR3] Australian Industry and Skills Committee (2021) National Industry Insights. Coal Mining. Accessed from: https://nationalindustryinsights.aisc.net.au/industries/mining-drilling-and-civil-infrastructure/coal-mining

[CR4] Avila S (2018). Environmental justice and the expanding geography of wind power conflicts. Sustain Sci.

[CR5] Bainton N, Kemp D, Lèbre E (2021). The energy-extractives nexus and the just transition. Sustain Dev.

[CR6] Beatty C, Fothergill S (1996). Labour market adjustment in areas of chronic industrial decline: the case of the UK coalfields. Reg Stud.

[CR7] Beatty C, Fothergill S (2020). The long shadow of job loss: Britain’s Older industrial towns in the 21st century. Front Sociol.

[CR8] Bennett K, Beynon H, Hudson R (2000) Coalfields regeneration: dealing with the consequences of industrial decline. Report by The Joseph Rowntree Foundation. https://www.jrf.org.uk/report/coalfields-regeneration-dealing-consequences-industrial-decline

[CR9] Berkhout F, Marcotullio P, Hanaoka T (2012). Understanding energy transitions. Sustain Sci.

[CR10] Błachowicz A et al (2021) Incorporating just transition strategies into developing countries NDCs and Covid-19 responses. Comparing insights from Ghana, Colombia, and Indonesia. Climate Strategies. Available on: https://climatestrategies.org/wp-content/uploads/2021/07/Incorporating-just-transitionstrategies-into-developing-countries-NDCs-and-Covid-19-responses.pdf

[CR11] Brauers H, Oei PY (2020). The political economy of coal in Poland: drivers and barriers for a shift away from fossil fuels. Energy Policy.

[CR12] Braunger I, Walk P (2022). Power in transitions: gendered power asymmetries in the United Kingdom and the United States coal transitions. Energy Res Soc Sci.

[CR13] Bryce R (2022) The iron law of electricity strikes again: Germany Re-Opens five lignite-fired power plants. https://www.forbes.com/sites/robertbryce/2022/10/28/the-iron-law-of-electricity-strikes-again-germany-re-opens-five-lignite-fired-power-plants/?sh=151eb5b93d0c

[CR14] Burke PJ, Best R, Jotzo F (2019). Closures of coal-fired power stations in Australia: local unemployment effects. Aust J Agric Resour Econ.

[CR15] Cai W, Aoyama Y (2018). Fragmented authorities, institutional misalignments, and challenges to renewable energy transition: a case study of wind power curtailment in China. Energy Res Soc Sci.

[CR16] Cai W, Mu Y, Wang C (2014). Distributional employment impacts of renewable and new energy—a case study of China. Renew Sustain Energy Rev.

[CR17] Caldecott B, Sartor O, Spencer T (2017) Lessons from previous ‘coal transitions’ high-level summary for decision-makers. IDDRI Report. Accessed from: https://www.iddri.org/en/publications-and-events/report/lessons-previous-coal-transitions

[CR18] Cao X (2017). Policy and regulatory responses to coal mine closure and coal resources consolidation for sustainability in Shanxi, China. J Clean Prod.

[CR19] Cao X, Kleit A, Liu C (2016). Why invest in wind energy? Career incentives and Chinese renewable energy politics. Energy Policy.

[CR20] Carley S, Konisky DM (2020). The justice and equity implications of the clean energy transition. Nat Energy.

[CR21] Carley S, Evans TP, Konisky DM (2018). Adaptation, culture, and the energy transition in American coal country. Energy Res Soc Sci.

[CR22] Carlisle K, Gruby RL (2019). Polycentric systems of governance: a theoretical model for the commons. Policy Stud J.

[CR23] Climate Action Tracker (2022) China. https://climateactiontracker.org/countries/china/

[CR24] Coalfield Regeneration Review Board. (2010) A Review of Coalfields Regeneration. https://assets.publishing.service.gov.uk/government/uploads/system/uploads/attachment_data/file/6295/1728082.pdf

[CR25] Colorado Department of Labor and Employment (2020) Colorado Just Transition Action Plan. https://cdle.colorado.gov/the-office-of-just-transition

[CR26] Cui RY, Hultman N, Cui D (2021). A plant-by-plant strategy for high-ambition coal power phaseout in China. Nat Commun.

[CR27] David M, Gross M (2019). Futurizing politics and the sustainability of real-world experiments: what role for innovation and exnovation in the German energy transition?. Sustain Sci.

[CR28] Delina LL, Sovacool BK (2018). Of temporality and plurality: an epistemic and governance agenda for accelerating just transitions for energy access and sustainable development. Curr Opin Environ Sustain.

[CR29] Della Bosca H, Gillespie J (2018). The coal story: generational coal mining communities and strategies of energy transition in Australia. Energy Policy.

[CR30] Department of Economic and Social Affairs, United Nations (2021) Enabling SDGs through inclusive, just energy transitions. https://www.un.org/sites/un2.un.org/files/2021-twg_3-exesummarie-062321.pdf

[CR31] Dezem V (2022) Germany Bolsters coal-fired power to meet winter power demand. https://www.bloomberg.com/news/olands/2022-10-21/germany-bolsters-coal-fired-power-to-meet-winter-power-demand?leadSource=uverify%20wall

[CR32] Diluiso F, Walk P, Manych N (2021). Coal transitions-part 1: a systematic map and review of case study learnings from regional, national, and local coal phase-out experiences. Environ Res Lett.

[CR33] Doering H (2014). Competing visions of community: empowerment and abandonment in the governance of coalfield regeneration: competing visions of community in the Kent Coalfield. Int J Urban Reg Res.

[CR34] Dong H, Liu Y, Zhao Z (2022). Carbon neutrality commitment for China: from vision to action. Sustain Sci.

[CR35] Downing TE, Shi G, Zaman M (2021). Improving post-relocation support for people resettled by infrastructure development. Impact Assess Project Apprais.

[CR36] Duan H, Zhou S, Jiang K (2021). Assessing China’s efforts to pursue the 1.5 C warming limit. Science.

[CR37] European Commission (2020) Governance of transitions: Design of governance structures and stakeholder engagement processes for coal regions in transition. Retrieved from https://ec.europa.eu/energy/topics/oil-gas-and-coal/EU-coal-regions/resources/governance-transitions-toolkit_en

[CR38] European Commission (2022a) The just transition mechanism: making sure no ”one is left behind. From https://ec.europa.eu/info/strategy/polandas-2019-2024/european-green-deal/finance-and-green-deal/just-transition-mechanism_en

[CR39] European Commission (2022b) Joint Statement by the Government of the Republic of Indonesia and International Partners Group members on the Indonesia Just Energy Transition Plan. https://ec.europa.eu/commission/presscorner/detail/en/STATEMENT_22_6892

[CR40] Fraser N (1995). From redistribution to recognition? Dilemmas of justice in a ‘post-socialist’ age. New Left Rev I.

[CR41] Furnaro A, Herpich P, Brauers H, et al (2021) German Just Transition: A Review of Public Policies to Assist German Coal Communities in Transition. https://www.edf.org/sites/default/files/documents/German%20Just%20Transition%20Case%20Study_0.pdf

[CR42] Galvin R, Healy N (2020). The Green New Deal in the United States: what it is and how to pay for it. Energy Res Soc Sci.

[CR43] García-Muros X, Morris J, Paltsev SJ (2022) Toward a just energy transition: a distributional analysis of low-carbon policies in the USA. Energy Econ 105

[CR44] Global Energy Monitor (2022) https://globalenergymonitor.org/projects/global-coal-mine-tracker/summary-tables/

[CR45] Glyn A, Machin S (1997). Colliery closures and the decline of the UK coal industry. Br J Ind Relat.

[CR46] Goch S (2002). Betterment without airs: social, cultural, and political consequences of de-industrialization in the Ruhr. Int Rev Soc Hist.

[CR47] Halper E (2022) Fossil fuel projects were stalled a year ago. Now they’re making a comeback. https://www.washingtonpost.com/business/2022d/11/03/fossil-fuel-cop27-russia/

[CR48] He G, Lin J, Zhang Y (2020). Enabling a rapid and just transition away from coal in China. One Earth.

[CR49] Heffron RJ (2021). What is the “just transition”? Achieving a just transition to a low-carbon economy.

[CR50] Heffron RJ, McCauley D (2018). What is the ‘just transition’?. Geoforum.

[CR51] Heffron RJ, McCauley D (2022). The ‘just transition’ threat to our Energy and Climate 2030 targets. Energy Policy.

[CR52] Hermwille L, Sanderink L (2019). Make fossil fuels great again? The Paris Agreement, Trump, and the US fossil fuel industry. Glob Environ Polit.

[CR53] Hielscher S, Wittmayer JM, Dańkowska A (2022). Social movements in energy transitions: the politics of fossil fuel energy pathways in the United Kingdom, the Netherlands and Poland. Extr Ind Soc.

[CR54] Hu Z (2020). When energy justice encounters authoritarian environmentalism: the case of clean heating energy transitions in rural China. Energy Res Soc Sci.

[CR55] Huang P, Liu Y (2021). Toward just energy transitions in authoritarian regimes: indirect participation and adaptive governance. J Environ Plann Manag.

[CR56] Huang P, Castán Broto V, Westman L (2020). Emerging dynamics of public participation in climate governance: a case study of solar energy application in Shenzhen China. Environ Policy Gov.

[CR57] Huang P, Westman L, Castan Broto V (2021). A culture-led approach to understanding energy transitions in China: the correlative epistemology. Trans Inst Br Geogr.

[CR58] IEA (2021) An energy sector roadmap to carbon neutrality in China. https://www.iea.org/reports/an-energy-sector-roadmap-to-carbon-neutrality-in-china

[CR59] International Climate Initiative (2019) Social and Economic Co-Benefits of Renewable Energy for South Africa. https://www.iass-potsdam.de/sites/default/files/2019-04/COBENEFITS_SA_Results_BETD_190405.pdf

[CR60] Isoaho K, Markard J (2020). The politics of technology decline: discursive struggles over coal phase-out in the UK(sic)(sic)(sic)Palabras Clave. Rev Policy Res.

[CR61] Jaramillo E (2022) China’s Hukou Reform in 2022e: Do They Mean it this Time? https://www.csis.org/blogs/new-perspectives-asia/chinas-hukou-reform-2022-do-they-mean-it-time0#:~:text=The%20hukou%20system%20codifies%20various,citizens%20can%20receive%20public%20services

[CR62] Jarvis S, Deschenes O, Jha A (2022). The private and external costs of Germany’s nuclear phase-out. J Eur Econ Assoc.

[CR63] Jenkins K, Sovacool B, Błachowicz A (2020). Politicising the just transition: linking global climate policy, nationally determined contributions and targeted research agendas. Geoforum.

[CR64] Jennings N, Fecht D, De Matteis S (2020). Mapping the co-benefits of climate change action to issues of public concern in the UK: a narrative review. Lancet Planet Health.

[CR65] Johnstone P, Hielscher S (2017). Phasing out coal, sustaining coal communities? Living with technological decline in sustainability pathways. Extr Ind Soc.

[CR66] Jotzo F, Mazouz S, Wiseman J (2018) Coal transition in Australia: an overview of issues. Centre for Climate and Energy Policy Working Papers, Crawford School of Public Policy.

[CR67] Kang K (2016). Policy influence and private returns from lobbying in the energy sector. Rev Econ Stud.

[CR68] Kinkartz S (2022) Germany’s energy U-turn: Coal instead of gas. www.htolandaw.dw.com/en/germanys-energy-u-turn-coal-instead-of-gas/a-62709160

[CR69] Kowalska IJ (2015). Challenges for long-term industry restructuring in the Upper Silesian Coal Basin: what has Polish coal mining achieved and failed from a twenty-year perspective?. Resour Policy.

[CR70] Krawchenko TA, Gordon M (2021). How do we manage a just transition? A comparative review of national and regional just transition initiatives. Sustainability.

[CR71] Li J (2010). Decarbonising power generation in China—is the answer blowing in the wind?. Renew Sustain Energy Rev.

[CR72] Li J, Krishnamurthy S, Roders AP (2020). Informing or consulting? Exploring community participation within urban heritage management in China. Habitat Int.

[CR73] Lin K, Lu X, Zhang J (2020). State-owned enterprises in China: a review of 40 years of research and practice. China J Account Res.

[CR74] Long H, Liu J, Tu C (2018). From state-controlled to polycentric governance in forest landscape restoration: the case of the ecological forest purchase program in Yong’an Municipality of China. Environ Manag.

[CR75] Loorbach D, Van der Brugge R, Taanman M (2008). Governance in the energy transition: practice of transition management in the Netherlands. Int J Environ Technol Manage.

[CR76] Lu J, Nemet G (2022). Market-led decline amidst intense politicization: coal in the United States. The Political Economy of Coal.

[CR77] Martewicz M, Skolimowski P (2022) People in Poland Are Burning Trash to Stay Warm This Winter. https://www.bloomberg.com/news/articles/2022-10-06/energy-crisis-people-in-poland-burn-trash-to-keep-warm-in-coal-shortage?leadSource=uverify%20wall#xj4y7vzkg

[CR78] Mayer A (2022). Support for displaced coal workers is popular and bipartisan in the United States: evidence from Western Colorado. Energy Res Soc Sci.

[CR79] McCauley DA, Heffron RJ, Stephan H (2013). Advancing energy justice: the triumvirate of tenets. Int Energy Law Rev.

[CR80] McCauley D, Ramasar V, Heffron R (2019). Energy justice in the transition to low carbon energy systems: exploring key themes in interdisciplinary research. Appl Energy.

[CR81] Morris AC, Kaufman N, Doshi S (2019) The risk of fiscal collapse in coal-reliant communities. https://energypolicy.columbia.edu/sites/default/files/file-uploads/RiskofFiscalCollapseinCoalReliantCommunities-CGEP_Report_072619.pdf

[CR82] Murray R, Baldwin J, Ridgway K (2005). Socio-economic decline and adaptation: South Yorkshire’s Former Coalfields. Local Econ.

[CR83] National Reform Programme (2022) 2022/2023 National Reform Programme. https://ec.europa.eu/info/sites/default/files/nrp_2022_poland_en_0.pdf

[CR84] NCSC (2021) China’s mid-century long-term low greenhouse gas emission development strategy [Unofficial translation]. https://unfccc.int/sites/default/files/resource/China%E2%80%99s%20Mid-Century%20Long-Term%20Low%20Greenhouse%20Gas%20Emission%20Development%20Strategy.pdf

[CR85] Oei PY, Brauers H, Herpich P (2020). Lessons from Germany’s hard coal mining phase-out: policies and transition from 1950 to 2018. Climate PoliCy.

[CR86] Oei PY, Hermann H, Herpich P (2020). Coal phase-out in Germany—implications and policies for affected regions. Energy.

[CR87] Ohlendorf N, Jakob M, Steckel JC (2022). The political economy of coal phase-out: exploring the actors, objectives, and contextual factors shaping policies in eight major coal countries. Energy Res Soc Sci.

[CR88] Pai S, Zerriffi H, Jewell J, Pathak J (2020). Solar has greater techno-economic resource suitability than wind for replacing coal mining jobs. Environ Res Lett.

[CR89] Peng S, Shi G, Zhang R (2021). Social stability risk assessment: status, trends and prospects—a case of land acquisition and resettlement in the hydropower sector. Impact Assess Project Apprais.

[CR90] PRI (2022) A just transition: achieving a people-centric green transition. http://finance.sina.com.cn/esg/investment/2022f-06-23/doc-imizmscu8341847.shtml

[CR91] Reitzenstein A, Popp R (2019) The German coal commission-A role model for transformative change? https://www.e3g.org/wpcontent/uploads/E3G_2019_Briefing_German_Coal_Commission.pdf

[CR92] Renn O, Marshall JP (2016). Coal, nuclear and renewable energy policies in Germany: from the 1950s to the “Energiewende”. Energy Policy.

[CR93] Schepelmann P, Kemp R, Schneidewind U, Brauch HG, Oswald-Spring Ú, Grin J, Scheffran J (2016). The eco-restructuring of the Ruhr District as an example of a managed transition. Handbook on sustainability transition and sustainable peace.

[CR94] Sicotte DM, Joyce KA, Hesse A (2022). Necessary, welcome or dreaded? Insights on low-carbon transitions from unionized energy workers in the United States. Energy Res Soc Sci.

[CR95] Smith BE (2015). Another place is possible? Labor geography, spatial dispossession, and gendered resistance in Central Appalachia. Ann Assoc Am Geogr.

[CR96] Snyder BF (2018). Vulnerability to decarbonization in hydrocarbon-intensive counties in the United States: a just transition to avoid post-industrial decay. Energy Res Soc Sci.

[CR97] Sovacool BK, Hess DJ, Cantoni R (2022). Conflicted transitions: exploring the actors, tactics, and outcomes of social opposition against energy infrastructure. Glob Environ Change.

[CR98] Steckel JC, Jakob M (2021). The political economy of coal: lessons learnt from 15 country case studies. World Dev Perspect.

[CR99] Stognief N, Walk P, Oei PY (2022). Political economy of climate and energy policies in the United Kingdom. The political economy of coal.

[CR100] Süsser D, Kannen A (2017). ‘Renewables? Yes, please!’: perceptions and assessment of community transition induced by renewable-energy projects in North Frisia. Sustain Sci.

[CR101] The German Coal Commission (2019a) A roadmap for a just transition from coal to renewables. https://coaltransitions.org/publications/the-german-coal-commission/

[CR102] The State Council of the People's Republic of China (2021) Outline of the People's Republic of China 14th Five-Year Plan for National Economic and Social Development and Long-Range Objectives for 2035. 中华人民共和国国民经济和社会发展第十四个五年规划和 2035 年远景目标纲要. http://www.gov.cn/xinwen/2021e-03/13/content_5592681.htm

[CR103] UNFCCC (2018) Just transition of the workforce, and the creation of decent work and quality jobs. https://unfccc.int/documents/226460

[CR104] Wang X, Lo K (2022). Political economy of just transition: Disparate impact of coal mine closure on state-owned and private coal workers in Inner Mongolia China. Energy Res Soc Sci.

[CR105] Wang Y, Zhang R, Worden S (2021). Public participation in environmental governance initiatives of chemical industrial parks. J Clean Prod.

[CR106] Ward M (2016) Regional Growth Fund. https://dera.ioe.ac.uk/27172/2/SN05874_Redacted.pdf

[CR107] Wilgosh B, Sorman A, Barcena I (2022). When two movements collide: learning from labour and environmental struggles for future Just Transitions. Futures.

[CR108] Wiseman J, Workman A, Fastenrath S et al (2020) After the Hazelwood coal fired power station closure: Latrobe Valley regional transition policies and outcomes 2017–2020 (CCEP Working Paper, Issue)

[CR109] Wishart R (2019). Class capacities and climate politics: Coal and conflict in the United States energy policy-planning network. Energy Res Soc Sci.

[CR110] World Bank (2022a) China: Country Climate and Development Report. Washington, DC. Accessed from: https://openknowledge.worldbank.org/bitstream/handle/10986/38136/FullReport.pdf

[CR111] World Bank (2022b) Just Transition for All: The World Bank Group's support to countries transitioning away from coal. https://www.worldbank.org/en/topic/extractiveindustries/justtransition

[CR112] World Energy & Climate Statistics (2022) Coal and lignite production. https://yearbook.enerdata.net/coal-lignite/coal-production-data.html

[CR113] Yenneti K, Day R, Golubchikov O (2016). Spatial justice and the land politics of renewables: dispossessing vulnerable communities through solar energy mega-projects. Geoforum.

[CR114] Yu H, Li S, Yu L (2022). The recent progress China has made in green mine construction, part II: typical examples of green mines. Int J Environ Res Public Health.

[CR115] Zhang S, Chen W (2022). Assessing the energy transition in China towards carbon neutrality with a probabilistic framework. Nat Commun.

[CR116] Zhang R, Worden S, Xu J (2022). Social stability risk assessment and economic competitiveness in China. Humanit Soc Sci Commun.

[CR117] Zhao F, Ma Y, Xi F (2020). Evaluating the sustainability of mine rehabilitation programs in China. Restor Ecol.

[CR118] Zheng S, Kahn ME, Sun W (2014). Incentives for China's urban mayors to mitigate pollution externalities: the role of the central government and public environmentalism. Reg Sci Urban Econ.

[CR119] Zhou B, Wang Q, Zhang C (2022). Central–local governance gaps: the evolving differentiation of climate policies in China. Sustain Sci.

